# The Impact of Nicotine Patches on Gingival and Oral Health: A Narrative Review

**DOI:** 10.7759/cureus.70571

**Published:** 2024-09-30

**Authors:** Haya Alayadi

**Affiliations:** 1 Dental Health Department, College of Applied Medical Sciences, King Saud University, Riyadh, SAU

**Keywords:** nicotine patches, oral health, periodontal diseases, smoking cessation, tobacco use cessation products

## Abstract

Smoking significantly impacts oral health, causing periodontal disease, oral cancer, tooth discoloration, halitosis, and impaired wound healing. Nicotine replacement therapy (NRT), particularly nicotine patches, is widely used for smoking cessation. This review evaluates the literature regarding nicotine patches and their implications for oral and gum health.

Nicotine patches deliver controlled nicotine doses transdermally, alleviating cravings and withdrawal symptoms. While nicotine can negatively affect oral health through vasoconstriction and reduced salivary flow, the overall impact of patches is generally positive compared to continued smoking. The primary benefit is the elimination of exposure to harmful chemicals and heat from cigarette smoke, significantly decreasing oral cancer risk and periodontal disease progression.

Nicotine patches are associated with improved periodontal treatment outcomes, better wound healing, and potential anti-inflammatory effects. They may also promote improved oral hygiene practices. However, limitations include uncertain long-term effects and potential side effects like xerostomia.

In conclusion, while nicotine patches may have some oral health effects, their use generally leads to significant improvements compared to continued smoking, making them valuable for promoting better oral health in individuals quitting smoking.

## Introduction and background

Smoking is a significant risk factor for various oral health problems [1]. The toxic chemicals in tobacco smoke can lead to issues ranging from cosmetic concerns like teeth staining to severe conditions such as oral cancer. Smokers are at an increased risk of developing periodontal disease, which can cause gum inflammation, bone loss, and eventually tooth loss [2]. Smoking also impairs the mouth's natural healing processes, making smokers more susceptible to infections and slower to recover from dental procedures [3]. Additionally, smoking contributes to reduced saliva production, leading to dry mouth and an increased risk of tooth decay [3].

Nicotine replacement therapy (NRT) is a widely accepted method for aiding smoking cessation. It works by providing controlled doses of nicotine without the harmful chemicals found in tobacco smoke [4]. NRT is available in various forms, including patches, gum, lozenges, nasal sprays, and inhalers. It is generally considered safe and can significantly increase the chances of successful smoking cessation when used correctly, especially when combined with behavioral support and counseling [5]. The aim of this review is to evaluate the literature regarding nicotine patches and their implications for oral and gum health.

## Review

Nicotine patches

Nicotine patches are a form of NRT designed to aid in smoking cessation [4]. These transdermal patches deliver controlled amounts of nicotine through the skin and into the bloodstream, helping to alleviate cravings and withdrawal symptoms associated with quitting smoking [6]. Nicotine patches are available in various strengths, typically starting with a higher dose that matches the user's current smoking habits and gradually decreasing over time [6]. 

Mechanism of Action

Nicotine patches work by delivering a controlled dose of nicotine through the skin and into the bloodstream [4]. This transdermal delivery system helps to reduce cravings and withdrawal symptoms associated with quitting smoking [4]. The patch provides a steady release of nicotine over an extended period, typically 16 or 24 hours, depending on the specific product [6].

Usage and Administration

Nicotine patches are typically applied once daily to clean, dry, hairless skin, often on the upper arm, chest, or back [7]. They come in various strengths, usually starting with a higher dose and gradually decreasing over time as part of a smoking cessation program [7]. It's important to rotate the application site daily to prevent skin irritation. Users are advised not to smoke while using the patch to avoid nicotine overdose.

Efficacy in Smoking Cessation

Nicotine patches have been shown to be an effective aid in smoking cessation [4,8]. Studies have consistently demonstrated that using nicotine patches can significantly increase the chances of successfully quitting smoking compared to placebo or no treatment [8]. When used as directed and combined with behavioral support or counseling, nicotine patches can approximately double the likelihood of long-term smoking abstinence [8].

Effects of smoking on oral health

While this review primarily focuses on oral health, it's important to note that smoking has wide-ranging systemic health implications beyond the oral cavity. Tobacco use is a major risk factor for numerous chronic diseases, including cardiovascular diseases, respiratory disorders, and various cancers [9]. Smoking damages nearly every organ in the body, significantly increasing the risk of coronary heart disease, stroke, and lung cancer [10]. It is also associated with increased risks of type 2 diabetes, rheumatoid arthritis, and reduced fertility [11]. The harmful effects of smoking extend to passive smokers as well, with second-hand smoke exposure linked to cardiovascular diseases and lung cancer in non-smoking adults and various health issues in children [12]. These systemic health risks underscore the importance of smoking cessation not only for oral health but for overall well-being.

Periodontal Disease

Smoking is a major risk factor for periodontal disease, significantly impacting the tissues supporting the teeth [8]. Smokers face up to six times higher risk of developing periodontal disease compared to non-smokers [13]. The mechanisms through which smoking contributes to periodontal disease are multifaceted. Nicotine causes vasoconstriction, reducing blood flow to the gums [14]. This impaired circulation hampers the delivery of vital oxygen and nutrients to periodontal tissues, rendering them more susceptible to infection and less capable of healing efficiently. Furthermore, smoking suppresses the immune system, compromising its ability to combat bacterial infections in the oral cavity [15].

Smoking alters the oral environment in ways that facilitate bacterial adhesion to teeth and gum surfaces, accelerating the formation of plaque and tartar [13]. Additionally, tobacco smoke impairs the function of fibroblasts, cells that play a crucial role in producing collagen and maintaining healthy gum tissue [16]. The combination of these factors leads to a more rapid progression of periodontal disease in smokers [2].

Oral Cancer

Smoking is the primary cause of oral cancer, dramatically elevating the risk of malignancies in the mouth, lips, tongue, and throat [2]. The carcinogenic compounds present in tobacco smoke, particularly nitrosamines and polycyclic aromatic hydrocarbons (PAHs), inflict damage on DNA and trigger cellular mutations, potentially leading to the development of cancerous lesions [17]. Smokers are 5-10 times more likely to develop oral cancer compared to non-smokers [18].

Smoking can induce the formation of precancerous conditions such as leukoplakia and erythroplakia, which have a high potential for malignant transformation [19]. Furthermore, smokers who develop oral cancer often face more challenging treatment outcomes and a higher risk of cancer recurrence compared to non-smokers [20].

Tooth Discoloration and Staining

Smoking is a significant contributor to tooth discoloration and staining, often resulting in an aesthetically unappealing yellowish or brownish tint to the teeth [21,22]. The tar and nicotine present in tobacco smoke can penetrate the enamel and dentin, causing intrinsic staining that proves challenging to remove through regular brushing [23]. Additionally, the heat from smoking induces thermal stress on the teeth, potentially making them more porous and thus more susceptible to staining [24].

Halitosis

Smoking is a significant contributor to halitosis, or chronic bad breath, due to several interrelated factors [25,26]. The habit reduces saliva production, creating a dry mouth environment that allows odor-causing bacteria to flourish [27,28]. Additionally, smoking alters the oral pH, creating favorable conditions for anaerobic bacteria that produce volatile sulfur compounds, the primary cause of bad breath [29].

Delayed Wound Healing

Smoking significantly impairs the oral cavity's ability to heal after dental procedures, extractions, or injuries [30]. The vasoconstrictive effect of nicotine decreases blood flow to the oral tissues, limiting the supply of oxygen and nutrients essential for healing [31]. Additionally, smoking suppresses the immune system, reducing the body's ability to fight infections and orchestrate the healing process effectively [31]. Tobacco smoke also interferes with collagen synthesis and remodeling, crucial processes in wound healing [18].

Nicotine patches and gingival health

Nicotine patches, while designed to aid in smoking cessation, can have some effects on gingival and oral health, though these are generally less severe than the effects of smoking itself [2]. The primary impact comes from the systemic delivery of nicotine, which can affect oral tissues even without direct contact with smoke. Nicotine, regardless of its delivery method, is a vasoconstrictor that can reduce blood flow to the gums [32]. This reduced blood supply may potentially impair gingival healing and decrease the effectiveness of the immune response to oral bacteria [33].

Some users might experience dry mouth (xerostomia) as a side effect of nicotine, which can increase the risk of dental caries and oral infections if prolonged [27]. However, it's important to note that these effects are typically milder than those caused by smoking, and the overall oral health benefits of quitting smoking generally outweigh any potential negative impacts of nicotine patch use [4].

Smoking not only impacts periodontal health and oral cancer risk but also contributes to other oral conditions. For instance, smokers are more prone to developing hairy tongue, a benign condition characterized by elongation and discoloration of filiform papillae on the tongue's surface [34]. Leukoplakia, a potentially premalignant condition presenting as white patches on the oral mucosa, is also strongly associated with smoking, with smokers having a sixfold higher risk compared to non-smokers [35]. Smoking can significantly alter taste and smell perception. Chronic exposure to tobacco smoke can lead to a reduction in the number and function of taste buds, resulting in decreased taste sensitivity, particularly for bitter tastes [36]. Similarly, olfactory function is often impaired in smokers, with studies showing a dose-dependent relationship between smoking intensity and olfactory dysfunction [37]. These sensory impairments can negatively impact quality of life and may persist even after smoking cessation, though some improvement is typically observed over time [3].

Direct Effects on Gingival Tissues

The direct effects of nicotine patches on gingival tissues are primarily mediated through the systemic delivery of nicotine, which reaches the oral cavity via the bloodstream [38]. One of the most significant impacts is vasoconstriction in the gingival tissues. Nicotine, being a potent vasoconstrictor, causes the blood vessels in the gums to narrow, reducing blood flow to these tissues [39,40]. This decreased blood supply can have several consequences. It may impair the delivery of nutrients and oxygen to the gingival cells, potentially compromising their health and function [33]. Furthermore, nicotine from patches can affect gingival tissues at a cellular level. It has been observed to alter the function of gingival fibroblasts, which are crucial for maintaining the health and integrity of gum tissues [41]. Nicotine can reduce the proliferation and attachment of these fibroblasts, potentially impacting the gums' ability to regenerate and repair themselves [42].

Comparison With Smoking's Effects on the Gingiva

Smoking has profound and well-documented negative effects on gingival health. The combustion products in cigarette smoke, including tar and numerous toxic chemicals, directly irritate the gingival tissues, leading to inflammation, reduced blood flow, and impaired immune response [43,44]. Smokers typically experience increased gingival recession, deeper periodontal pockets, and more rapid progression of periodontal disease compared to non-smokers [45].

In contrast, nicotine patches, while not entirely benign, have a considerably less harmful impact on gingival health. The primary effect of nicotine patches on the gingiva is through the systemic delivery of nicotine, which can cause some degree of vasoconstriction [32]. This may slightly reduce blood flow to the gingival tissues, potentially impacting their ability to fight infection and heal [39]. However, nicotine patches do not expose the gingiva to the direct irritants, heat, or toxic chemicals present in cigarette smoke [4].

Nicotine patches and dental health

Nicotine patches, while designed to aid in smoking cessation, can have both positive and negative impacts on dental health. On the positive side, by helping individuals quit smoking, nicotine patches indirectly contribute to improved oral health outcomes [4]. By facilitating smoking cessation, nicotine patches help reduce exposure to harmful chemicals in tobacco smoke that directly damage oral tissues [32]. This can lead to a decreased risk of tooth discoloration, gum disease, and oral cancer [46]. However, nicotine itself, even when delivered through patches, can have some effects on dental health. Nicotine is a vasoconstrictor, meaning it can reduce blood flow to the gums and other oral tissues [39]. This reduced blood supply may potentially impair gingival healing and decrease the effectiveness of the immune response to oral bacteria [33]. Some users of nicotine patches may experience dry mouth (xerostomia) as a side effect [47]. If prolonged, dry mouth can increase the risk of dental caries and oral infections due to reduced salivary flow, which normally helps in cleaning the mouth and neutralizing acids [48].

Effects on Tooth Enamel

Nicotine patches, while generally considered a safer alternative to smoking, may have some indirect effects on tooth enamel. The primary mechanism through which nicotine patches could potentially impact tooth enamel is through their influence on saliva production and composition [32]. Nicotine, regardless of its delivery method, can cause a reduction in saliva flow, leading to a condition known as xerostomia or dry mouth [27]. When saliva flow is reduced, the oral environment becomes more acidic, which can increase the risk of enamel demineralization and dental caries [40]. However, it's important to note that the effects of nicotine patches on tooth enamel are generally much less severe compared to the direct effects of smoking [4]. Unlike smoking, nicotine patches do not expose the teeth to the harmful chemicals and heat present in tobacco smoke, which can cause significant enamel discoloration and erosion [21].

Impact on Saliva Production and Composition

Nicotine patches, while designed to aid in smoking cessation, can have notable effects on saliva production. Nicotine, regardless of its delivery method, acts as a stimulant on the autonomic nervous system, which can influence salivary gland function [32]. Some studies have shown that nicotine can initially increase saliva production due to its stimulatory effects [27]. However, with prolonged use, nicotine has been associated with a decrease in salivary flow rate [49].

The composition of saliva can also be affected by nicotine delivered through patches. Nicotine has been shown to alter the protein content and enzymatic activity of saliva [50]. Studies have reported changes in the levels of certain salivary enzymes, such as amylase and lysozyme, which play crucial roles in oral health maintenance [51]. Additionally, nicotine can influence the pH of saliva, potentially making it more acidic [52].

Influence on Oral Microbiome

The oral microbiome, a complex ecosystem of microorganisms residing in the oral cavity, plays a crucial role in maintaining oral health [53]. While the direct impact of nicotine patches on the oral microbiome is less pronounced compared to smoking, the systemic delivery of nicotine can still influence this delicate balance. Nicotine, regardless of its delivery method, has been shown to affect the growth and virulence of certain oral bacteria [54]. Studies have indicated that nicotine can enhance the growth of periodontal pathogens such as *Porphyromonas gingivalis* and *Aggregatibacter actinomycetemcomitans*, potentially increasing the risk of periodontal disease [55,56]. The impact of nicotine patches on the oral microbiome is typically milder and less detrimental compared to the effects seen in active smokers [13]. Smoking introduces a myriad of harmful chemicals into the oral cavity, causing more dramatic shifts in the microbial community [57]. Nicotine patches, by contrast, deliver nicotine systemically without the accompanying smoke-related toxins [4].

Nicotine Patches and the Oral Mucosa

Nicotine patches, while not in direct contact with the oral mucosa, can still exert effects on this tissue through systemic nicotine delivery. The oral mucosa, comprising the lining of the oral cavity, is highly vascularized and sensitive to changes in blood composition, including the presence of nicotine [58]. When nicotine is absorbed through the skin from patches, it enters the bloodstream and circulates throughout the body, including the oral tissues [38]. One of the primary effects of nicotine on the oral mucosa is vasoconstriction, which can lead to reduced blood flow to these tissues [59].

The impact of nicotine patches on oral mucosa should also be considered in the context of smoking cessation. While nicotine itself may have some effects on the oral mucosa, the overall benefit of quitting smoking far outweighs these potential minor impacts [2]. Smoking exposes the oral mucosa to a multitude of harmful chemicals and carcinogens, leading to more severe and direct damage [60]. In contrast, nicotine patches eliminate this direct exposure, potentially allowing the oral mucosa to begin healing processes [61].

Effects on Oral Epithelial Cells

Nicotine, whether delivered through smoking or NRTs like patches, can have significant effects on oral epithelial cells. Studies have shown that nicotine can alter the proliferation and differentiation of oral epithelial cells [62]. At lower concentrations, nicotine has been observed to stimulate cell proliferation, potentially leading to hyperplasia of the oral epithelium. However, at higher concentrations, nicotine can induce cytotoxicity and apoptosis (programmed cell death) in these cells [63].

The effects of nicotine on oral epithelial cells extend beyond direct cellular impacts to influence their interaction with the oral environment. Nicotine has been shown to alter the permeability of the oral epithelium, potentially allowing for the increased penetration of toxins and pathogens [64]. This could contribute to an increased susceptibility to oral infections and diseases.

Impact on Oral Lesions and Precancerous Conditions

The impact of nicotine patches on oral lesions and precancerous conditions is a topic of significant interest in the field of oral health and smoking cessation. While smoking is a well-established risk factor for various oral lesions and precancerous conditions, the role of nicotine alone, as delivered through patches, is less clear-cut [2]. Nicotine, the primary addictive component in tobacco, has been shown to have some effects on cell proliferation and angiogenesis, which could potentially influence the development or progression of oral lesions [65].

Research suggests that the use of NRT, including patches, as part of a smoking cessation program may contribute to the regression of some oral lesions that were induced by smoking [66]. This is primarily due to the elimination of exposure to tobacco smoke, which contains numerous carcinogens and irritants. For instance, studies have shown that smoking cessation can lead to the regression of oral leukoplakia, a potentially precancerous condition [67].

Comparison With Smoking's Effects on the Oral Mucosa

Smoking has profound and detrimental effects on the oral mucosa, significantly more severe than those associated with nicotine patches [2,60]. The direct exposure of oral tissues to tobacco smoke leads to a range of harmful consequences. Smoking causes thermal damage to the oral epithelium, introduces numerous carcinogens, and exposes the mucosa to thousands of toxic chemicals [1]. This results in increased keratinization of the oral mucosa, reduced blood supply due to vasoconstriction, and suppression of the immune response in the oral cavity [60,68].

In contrast, nicotine patches deliver nicotine systemically without direct contact with the oral mucosa, resulting in considerably milder effects [28]. While nicotine itself can cause some vasoconstriction, potentially reducing blood flow to oral tissues, the absence of direct smoke exposure eliminates many of the more harmful effects associated with smoking [69]. Nicotine patches do not introduce carcinogens or cause thermal damage to the oral mucosa. They also do not lead to the increased keratinization or the development of precancerous lesions commonly seen in smokers [46]. The systemic delivery of nicotine through patches may cause some dry mouth (xerostomia), which could potentially increase the risk of oral infections if prolonged [70]. However, this effect is generally less severe and more manageable compared to the multifaceted damage caused by smoking. Overall, while nicotine patches are not entirely without oral health implications, their effects on the oral mucosa are significantly less harmful than those of smoking, making them a much safer alternative for individuals trying to quit tobacco use [4].

Potential benefits of nicotine patches for oral health

Nicotine patches, as a smoking cessation aid, offer significant potential benefits for oral health primarily by helping individuals quit smoking [2,71]. The most immediate benefit is the elimination of direct exposure of oral tissues to the harmful chemicals and heat present in cigarette smoke. By avoiding this exposure, users of nicotine patches can significantly reduce their risk of developing oral cancer, one of the most severe consequences of smoking. Studies have shown that the risk of oral cancer begins to decrease soon after smoking cessation, with a 50% reduction in risk within 3-5 years of quitting [72]. Additionally, the use of nicotine patches can lead to improvements in periodontal health. Smoking is a major risk factor for periodontal disease, and cessation has been associated with reduced progression of periodontal attachment loss and improved outcomes of periodontal treatments [61].

Furthermore, nicotine patches can contribute to better oral health outcomes by mitigating some of the negative effects of smoking withdrawal on oral tissues. During smoking cessation, individuals often experience increased appetite and snacking, which can lead to an increased risk of dental caries [21]. Nicotine patches, by helping to control cravings and withdrawal symptoms, may help regulate these behaviors. Moreover, smoking cessation facilitated by nicotine patches can lead to improved wound healing in the oral cavity, which is particularly beneficial for individuals undergoing dental surgical procedures [30].

Reduced Exposure to Harmful Tobacco Compounds

One of the primary benefits of using nicotine patches for smoking cessation is the significant reduction in exposure to harmful tobacco compounds. Cigarette smoke contains over 7,000 chemicals, including at least 70 known carcinogens [73]. These include PAHs, tobacco-specific nitrosamines (TSNAs), volatile organic compounds (VOCs), and heavy metals such as cadmium and lead [74]. When individuals switch from smoking to using nicotine patches, they eliminate their exposure to these harmful compounds. This reduction is particularly beneficial for oral health, as the oral cavity is the first point of contact for cigarette smoke [2].

Nicotine patches deliver only nicotine, without the myriad of other harmful substances found in tobacco smoke. While nicotine itself is not without physiological effects, it is not the primary cause of smoking-related diseases [75]. The use of nicotine patches allows individuals to manage their nicotine cravings without exposure to the more harmful components of tobacco smoke. This selective delivery is particularly important for oral health, as it eliminates exposure to substances like acrolein and formaldehyde, which are highly irritating to oral tissues and can contribute to inflammation and tissue damage [76].

Improved Oral Hygiene Practices During Cessation

Smoking cessation often serves as a catalyst for overall lifestyle improvements, including enhanced oral hygiene practices. As individuals quit smoking, they frequently report an increased awareness of their oral health and a renewed motivation to maintain good oral hygiene [77]. This heightened consciousness can be attributed to several factors. Firstly, the absence of smoking allows for a noticeable improvement in taste and smell sensations, which can make individuals more aware of their oral environment [36]. This sensory enhancement often leads to a desire for a cleaner, fresher mouth, encouraging more frequent and thorough brushing and flossing.

The process of quitting smoking, particularly when using aids like nicotine patches, often involves regular interaction with healthcare professionals, including dental care providers [78]. These interactions present opportunities for education about proper oral hygiene techniques and the importance of regular dental care. Dental professionals can use this time to provide tailored advice on addressing specific oral health issues that may have developed due to smoking, such as gum disease or staining [79].

Potential Anti-inflammatory Effects

Nicotine, the primary active compound delivered by nicotine patches, has been found to exhibit complex effects on inflammation, including some potential anti-inflammatory properties [80,81]. This is particularly relevant in the context of oral health, where chronic inflammation plays a significant role in conditions such as periodontal disease. The anti-inflammatory effects of nicotine are primarily mediated through its interaction with nicotinic acetylcholine receptors (nAChRs), particularly the α7 subtype, which are expressed on various immune cells including macrophages and neutrophils [82]. Activation of these receptors has been shown to suppress the production of pro-inflammatory cytokines such as tumor necrosis factor-α (TNF-α), interleukin-1β (IL-1β), and interleukin-6 (IL-6) [83].

It is crucial to note that the anti-inflammatory effects of nicotine are dose-dependent and context-specific. While low to moderate doses of nicotine have demonstrated anti-inflammatory properties in some studies, high doses or chronic exposure can actually promote inflammation [84]. In the oral cavity, the potential anti-inflammatory effects of nicotine delivered through patches must be weighed against its other effects, such as vasoconstriction, which can impair immune function and healing [85].

Limitations and considerations

The use of nicotine patches as a smoking cessation aid presents several limitations and considerations, particularly in relation to oral health. One significant factor is the distinction between short-term and long-term effects. In the short term, nicotine patches can help manage withdrawal symptoms and reduce the immediate negative impacts of smoking cessation on oral health, such as increased snacking that may lead to dental caries [13]. However, the long-term effects of prolonged nicotine patch use on oral tissues are less well understood. While nicotine itself has been associated with reduced gingival blood flow and potential impairment of fibroblast function, these effects are generally considered less severe than those of smoking [86].

The interaction of nicotine patches with other oral health factors is another crucial consideration. For instance, individuals with pre-existing periodontal disease or a history of oral cancer may require special monitoring when using nicotine patches, as the systemic delivery of nicotine could potentially influence disease progression or recurrence [2]. Moreover, nicotine patches do not address the behavioral aspects of smoking that can affect oral health, such as the hand-to-mouth action that smokers often replace with increased snacking or the consumption of sugary foods. This behavioral change may increase the risks of tooth decay and erosion [87].

Clinical implications

Dental professionals play a crucial role in supporting patients' smoking cessation efforts, including those using nicotine patches. It's recommended that dental practitioners incorporate tobacco cessation counseling into their routine care, utilizing the 5A approach: ask about tobacco use, advise to quit, assess willingness to quit, assist in quit attempt, and arrange follow-up [6]. When patients are using nicotine patches, dental professionals should be aware of potential oral health implications and adjust their treatment plans accordingly. For instance, due to the vasoconstrictive effects of nicotine, caution may be needed when using local anesthetics containing vasoconstrictors [32].

Monitoring oral health during nicotine patch use is an essential aspect of comprehensive dental care for patients quitting smoking. Dental professionals should conduct thorough oral examinations at regular intervals, paying particular attention to mucosal changes, periodontal health, and salivary flow [88,89]. It's advisable to establish a baseline of the patient's oral health status before they begin using nicotine patches and track any changes over time. This monitoring should include periodontal probing, assessment of plaque and calculus levels, and evaluation of any pre-existing oral conditions that may be affected by nicotine patch use [61].

Future research directions

Future investigations into NRT and oral health should prioritize long-term studies examining long-term oral health effects [2]. Longitudinal research could elucidate the impacts of various NRT approaches on periodontal health, oral mucosa condition, and salivary gland function across time [61,90]. Researchers should explore the enduring effects of persistent low-dose nicotine exposure on oral structures, including its influence on healing processes, immune responses, and oral microbial communities [32]. Additionally, comparative analyses of diverse NRT methods' oral health implications are essential to determine the most advantageous or least detrimental options [4,91].

Another critical research avenue is the development of individualized NRT strategies tailored to oral health considerations [6]. This may encompass studying how personal characteristics interact with different NRT methods to shape oral health outcomes [92]. Investigations could also assess whether specific NRT approaches are better suited for patients with particular oral health issues [93]. Moreover, research into identifying biomarkers that predict individual responses to various NRT methods could prove highly beneficial [5].

## Conclusions

Nicotine patches, as a smoking cessation aid, present a complex profile of effects on oral health, but their overall impact is generally positive compared to continued smoking. The primary benefits include the elimination of exposure to harmful chemicals and heat from cigarette smoke, a significant decrease in oral cancer risk and periodontal disease progression, improved periodontal treatment outcomes, better wound healing in the oral cavity, and potential anti-inflammatory effects. While nicotine patches may cause some side effects like temporary xerostomia (dry mouth) and vasoconstriction, these are generally milder and less detrimental than the multiple harmful effects of smoking on oral health. The use of nicotine patches as part of a smoking cessation program can lead to significant improvements in oral health outcomes, making them a valuable tool for promoting better oral health in individuals quitting smoking.

Despite the overall positive impact, continued research and clinical vigilance are crucial. Areas requiring further investigation include long-term effects on oral tissues, interactions with dental materials, and optimal duration of use. Clinicians should monitor the oral health of patients using nicotine patches, particularly those with pre-existing conditions. Regular dental check-ups and open communication between patients and healthcare providers are essential to maximize benefits while minimizing potential risks. In conclusion, while nicotine patches are not without their challenges, they represent an important strategy in improving oral health outcomes for individuals seeking to quit smoking, balancing the complex interplay between smoking cessation and oral health management.

## Appendices

Search strategy and quality assessment criteria

This narrative review employed a comprehensive search strategy to identify relevant literature on the impact of nicotine patches on gingival and oral health. Electronic databases including PubMed, Cochrane Library, and EMBASE were searched using keywords such as "nicotine replacement therapy," "nicotine patches," "oral health," and "periodontal disease." The search was limited to English-language articles published between 1980 and 2023. Studies were included if they addressed the effects of nicotine patches on any aspect of oral health, including periodontal disease, oral cancer, and salivary function. The quality of included studies was assessed using the Newcastle-Ottawa Scale for observational studies and the Cochrane Risk of Bias tool for randomized controlled trials. Priority was given to systematic reviews, meta-analyses, and well-designed clinical trials. Case reports and studies with significant methodological flaws were excluded. The author had screened titles and abstracts, and this rigorous approach ensured the inclusion of high-quality evidence to support the findings and conclusions of this review.
